# Intracellular Proteolytic Systems in Alcohol-Induced Tissue Injury

**Published:** 2003

**Authors:** Terrence M. Donohue, Natalia A. Osna

**Affiliations:** Terrence M. Donohue, Jr., Ph.D., is a research scientist at the Omaha VA Medical Center and an associate professor in the Departments of Internal Medicine and Biochemistry/Molecular Biology at the University of Nebraska Medical Center, Omaha. Natalia A. Osna, M.D., Ph.D., is a researcher at the Omaha VA Medical Center and an instructor in the Department of Internal Medicine, University of Nebraska Medical Center, Omaha. Studies mentioned in this review that were performed by the authors were supported by National Institute on Alcohol Abuse and Alcoholism grant AA–09384 and by Medical Research funds from the Department of Veterans Affairs

**Keywords:** ethanol metabolism, protein metabolism disorder, biochemical reaction property, tissue, injury, inflammation, alcohol dehydrogenases, cytochrome P450 2E1, AODR (alcohol and other drug related) injury, proteolysis, protease inhibitors, cytolysis, apoptosis, lysosome, coenzymes, physiological AODE (alcohol and other drug effects), ubiquitin-proteasome system

## Abstract

The body constantly produces proteins and degrades proteins that are no longer needed or are defective. The process of protein breakdown, called proteolysis, is essential to cell survival. Numerous proteolytic systems exist in mammalian cells, the most important of which are the lysosomes, the ubiquitin–proteasome pathway, and enzymes called calpains. Lysosomes are small cell components that contain specific enzymes (i.e., proteases) which break down proteins. Alcohol interferes with the formation and activity of lysosomes and thus may contribute to protein accumulation in the liver, which can have harmful effects on that organ. In the ubiquitin–proteasome pathway, proteins that are to be degraded are first marked by the addition of ubiquitin molecules and then broken down by large protein complexes called proteasomes. Alcohol impairs this proteolytic system through several mechanisms, possibly leading to inflammation and even cell death. Calpains are proteases that are involved in several physiological processes, including the breakdown of proteins that give cells their shape and stability. In contrast to the lysosomal and ubiquitin–proteasome systems, calpains in brain cells are activated by alcohol, to potentially detrimental effect.

In all cells, the production and degradation of proteins, commonly known as protein turnover, is a constant, ongoing process that is crucial for tissue renewal. A well-nourished person synthesizes nearly 1 pound of protein per day. This protein gain is balanced by an equal amount of proteins that are broken down into their building blocks, the amino acids. This cycle of protein turnover is a necessary component of cell survival and repair because it ensures that damaged proteins are degraded and that all proteins needed by the cells for a variety of functions are available at the right time and in the right amounts.

Compared with the intense interest of researchers in protein synthesis, protein degradation—also known as protein catabolism or proteolysis—was for years considered a backwater area of scientific investigation. This neglect resulted largely from the assumption that, in contrast to the relative complexity of protein synthesis, protein degradation consisted of a random array of biochemical reactions that were facilitated (i.e., catalyzed) by certain enzymes and only involved breaking apart the chemical peptide bonds that hold amino acids together. Research over the last 30 years has revealed, however, that protein degradation occurs in a highly coordinated, specific manner through multiple systems which rival the complexity of protein synthesis. Moreover, all protein degradation systems are tightly regulated in order to maintain a “steady state” level of intracellular proteins. If this steady state is disrupted by metabolic disturbances, by the presence of surplus reactive molecules such as free radicals, or by toxic agents such as alcohol, cell injury and sometimes cell death can occur (Mehlhase and Grune 2002).

This article describes the significance of protein degradation for cell metabolism, introduces the major systems involved in this process, and explores ways in which alcohol consumption can disrupt protein catabolism, thereby bringing about tissue injury in the liver as well as other organs.

## The Metabolic Importance of Protein Degradation

**Figure 1:**
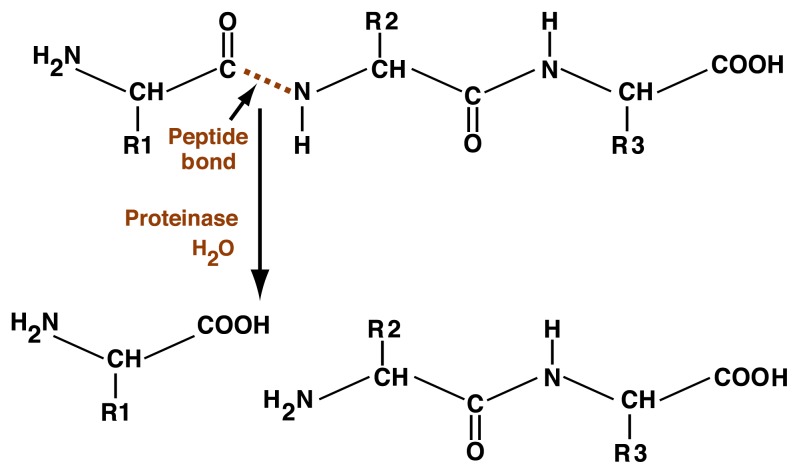
Schematic representation of proteolysis. The figure shows the basic chemical structure of a protein’s two amino acids, which are connected by a peptide bond (represented by the dotted line in the figure). It is at the peptide bond that the proteinase, with the addition of water (H_2_O), cuts the protein into two fragments. NOTE: R1, R2, and R3 represent side chains that are unique to each amino acid.

Proteins are degraded by a type of chemical reaction known as hydrolysis— a simple reaction in which a protein is “cut” at one or more peptide bonds by the addition of water, either to generate smaller protein fragments (i.e., peptides) or to completely break down the protein into individual amino acids (see figure 1). Enzymes that catalyze protein breakdown are called proteinases. Each proteinase recognizes the chemical structures of certain specific amino acids and then catalyzes the breaking of the peptide bond.

Proteolysis is essential for cell survival for several reasons. First, usable amino acids derived from protein degradation are recycled for the production of new proteins or are converted into vital energy molecules, such as glucose. Second, many newly synthesized proteins, as part of their maturation, must undergo partial degradation (i.e., removal of peptide fragments) to achieve their intended biological functions or be directed to their appropriate place in the cell. Third, proteins that are damaged by reactions with small highly reactive oxygen-containing molecules (i.e., reactive oxygen species), or that undergo other types of chemical modification, must be degraded to prevent their accumulation, which could prove toxic to the cell.

Fourth, proteolysis regulates the life span of proteins. Individual proteins possess remarkably consistent half-lives that range from several minutes for short-lived proteins to several days for long-lived proteins. The half-life of a protein is often related to the protein’s function. For example, proteins that perform control functions, such as the regulation of gene activity, usually are functional for only short periods and then are degraded quickly. Conversely, proteins that are needed constantly because they have catalytic or structural roles typically are long-lived. These long-lived proteins are more resistant to degradation than short-lived proteins. To ensure the accurate functioning of all cells, it is important to maintain the appropriate half-lives of all proteins through tightly regulated proteolysis.

## Intracellular Proteolytic Systems

Cells have well over 200 different proteinases that, either by themselves or as part of a larger proteolytic system composed of several enzymes, degrade proteins (see the accompanying table for a summary of these proteinases and systems). This article focuses on three major proteolytic systems known to be affected by alcohol consumption—the lysosome, the ubiquitin–proteasome system, and the calpains. Because of the recent intense interest in the ubiquitin–proteasome pathway and its role in many cell processes and diseases, this system and its role in alcohol-induced disease will be described in more detail than the other systems.

### The Lysosome System

Degradation in cell structures called lysosomes is the best-known means of protein disposal. Lysosomes are particles that are enclosed by a membrane and have an acidic interior, which provides a favorable environment for the many proteinases that reside within. Most cells contain numerous lysosomes whose numbers fluctuate with the cell’s nutritional state. Lysosomes primarily are specialized for breaking down proteins that enter the cell by a process called endocytosis.^1^ However, lysosomes also degrade intracellular proteins “in bulk” when large amounts of the fluid and particles filling the cell (i.e., the cytoplasm) become enclosed by intracellular membranes in a process called autophagy. Autophagy occurs during times of fasting or starvation, when cells are in greatest need of fuel to maintain their metabolism. This fuel is provided in part by amino acids derived from protein degradation. For example, fasting for 48 hours can result in the loss of 20 percent of liver proteins because of enhanced autophagy and accelerated protein breakdown in the lysosomes (Donohue et al. 1994). In addition to autophagy, other specific mechanisms exist through which intracellular proteins can enter the lysosomes and be degraded (for a detailed review, see Cuervo and Dice 2000).

#### Effects of Alcohol Consumption on the Lysosomes

Strong evidence suggests that alcohol consumption can alter the activities of lysosomes. One effect of alcohol consumption is an elevation in lysosomal pH (Kharbanda et al. 1997). This shift to alkalinity reduces the activities of lysosomal proteinases and results in less-than-optimal protein hydrolysis in the lysosome. Furthermore, the reduction in protein degradation contributes to excessive protein accumulation, which has been observed in livers of human alcoholics and alcohol-fed laboratory animals. Such increases in protein levels appear to occur specifically in the liver, possibly because this organ metabolizes 90 percent of the alcohol that is consumed. Abnormal protein accumulation is potentially toxic to cells because some of these accumulated proteins are damaged and/or can form protein aggregates, which can alter cellular metabolism and reduce cell viability.

**Figure 2:**
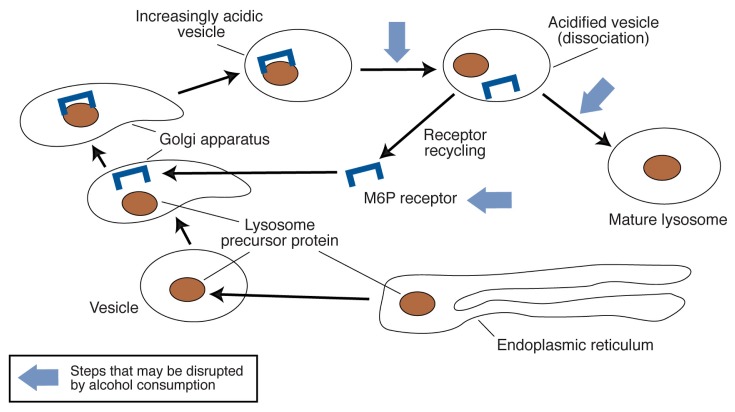
Schematic representation of the assembly of lysosomes in the cell. The figure depicts the processing and transport of one lysosome enzyme precursor protein. This precursor is formed in the cellular compartment called the endoplasmic reticulum, then moved via a small, membrane-enclosed “bubble” (i.e., a vesicle) to another membrane-enclosed compartment called the Golgi apparatus. There, the precursor is modified so it can be recognized by a transport protein, the mannose-6-phosphate (M6P) receptor (represented in the figure by a bracket). The M6P receptor binds to the precursor and moves it into another vesicle. The inside of this vesicle becomes gradually more acidic (i.e., the pH becomes lower) until the M6P receptor–enzyme precursor complex breaks apart. At this point, the released M6P receptor can be recycled to bind other lysosomal enzyme precursors, whereas the precursor-containing vesicle undergoes further maturation to become a lysosome. Alcohol consumption can disrupt lysosome assembly by decreasing the number of M6P receptors available for sending the precursor proteins to the lysosomes, preventing the acidification of lysosome precursor vesicles as well as lysosomes, and preventing the maturation of the lysosome enzyme precursor.

Alcohol and the products generated during alcohol metabolism also can interfere with the assembly of lysosomes, during which newly synthesized proteinases must be directed to the newly formed lysosomes. This process involves an elaborate series of steps that depend on proper amounts of functioning proteins called receptors which help transport lysosomal components (see figure 2). Alcohol metabolism in the liver can impede these steps by generating acetaldehyde and other highly reactive molecules that are believed to disrupt the processing (maturation) of proteins as well as their movement (trafficking) from the place where they are formed to their final destinations both inside and outside the cell. Moreover, researchers have found that alcohol consumption retards the maturation and processing of the precursors of proteinases destined for placement in the lysosomes (Kharbanda et al. 1996). One of the receptor molecules transporting lysosome components is the mannose-6-phosphate receptor (M6P receptor) (see figure 2), which recognizes and binds proteinase precursors during their transit to the lysosome. Alcohol consumption adversely affects the synthesis of these receptors (Haorah et al. 2002, 2003). As a consequence, fewer M6P receptors are present in the primary liver cells (i.e., hepatocytes) of alcohol-fed animals than in hepatocytes of animals that have not been fed alcohol (Haorah et al. 2002, 2003). A shortage of receptors eventually can lead to the formation of lysosomes that contain too few proteinases and therefore are ill equipped for adequate proteolysis.

Moreover, faulty processing and trafficking of lysosomal precursors and other components caused by alcohol consumption may lead to the assembly of lysosomes that are structurally defective and therefore more prone to leaks or rupture. Large-scale rupture of lysosomes within a cell can lead to cell death because cellular components that are normally protected by the lysosomal membrane become exposed to released lysosomal proteinases.

**Table:** Summary of Cellular Proteolytic Systems*

System	Location	Major Specificity/Function	Alcohol Effects
Lysosome**	Cytoplasm	Degrades intracellular proteins and proteins taken up by cellular endocytosis	Impairs activity, formation, and structural integrity of lysosomes
Ubiquitin– proteasome**	Cytoplasm, nucleus	Degrades most intracellular proteins as well as damaged proteins	Inhibits proteasome activity; leads to accumulation of ubiquitin–protein conjugates
Calpains**	Cytoplasm	Degrade cytoskeletal proteins	Activates calpains and leads to aberrant hydrolysis of cellular proteins
Mitochondrial proteinases	Mitochondria	Remove oxidatively modified mitochondrial proteins; partially degrade imported mitochondrial proteins	No known effect
Membrane proteinases	Endoplasmic reticulum, plasma membrane, mitochondria	Partially degrade secretory, endocytosed, and imported proteins; degrade newly synthesized faulty proteins	No known effect

* For definitions of terms used in this table, see the Glossary on page 331.

** Indicates that the system is described in greater detail in this article.

### The Ubiquitin–Proteasome System

The ubiquitin–proteasome system is now considered the major system involved in protein degradation within cells (for reviews of this system, see Bochtler et al. 1999; DeMartino and Slaughter 1999; Hershko and Ciechanover 1998; Hershko et al. 2000; Voges et al. 1999). The two main components of this system are (1) three enzymes that add a small protein called ubiquitin onto substrate proteins destined for degradation, and (2) the proteasome, a rather large cellular particle composed of several smaller protein subunits, which executes the actual proteolysis. By degrading short-lived regulatory proteins, the ubiquitin–proteasome system controls basic cellular processes such as cell division, cell signaling, and regulation of gene activity. The system also removes misfolded, damaged proteins, and in certain immune cells it breaks foreign proteins down into pieces called antigenic peptides, which can then be transported to the cell surface to induce an immune response. Many intracellular proteins now have been identified that are degraded by the ubiquitin– proteasome system. Among them are regulatory proteins; proteins involved in apoptosis, or programmed cell death (discussed in the section “The Ubiquitin– Proteasome System and Alcohol-Induced Cell Death”); and proteins involved in signaling processes in the cell (Ulrich 2002).

Most proteins destined for degradation by proteasomes first are linked to ubiquitin, a small protein consisting of 76 amino acids. This linkage process, called ubiquitylation, requires three classes of enzymes: (1) E1 enzymes, which activate the ubiquitin, (2) E2 enzymes, which bind to the ubiquitin molecule, and (3) E3 enzymes, which transfer the ubiquitin molecule to the target protein. In most cases, a chain of at least four ubiquitin molecules must be attached to the target protein. The resulting ubiquitin-linked protein, commonly called a ubiquitin–protein conjugate, then can be recognized and degraded into peptides by the proteasome.

**Figure 3:**
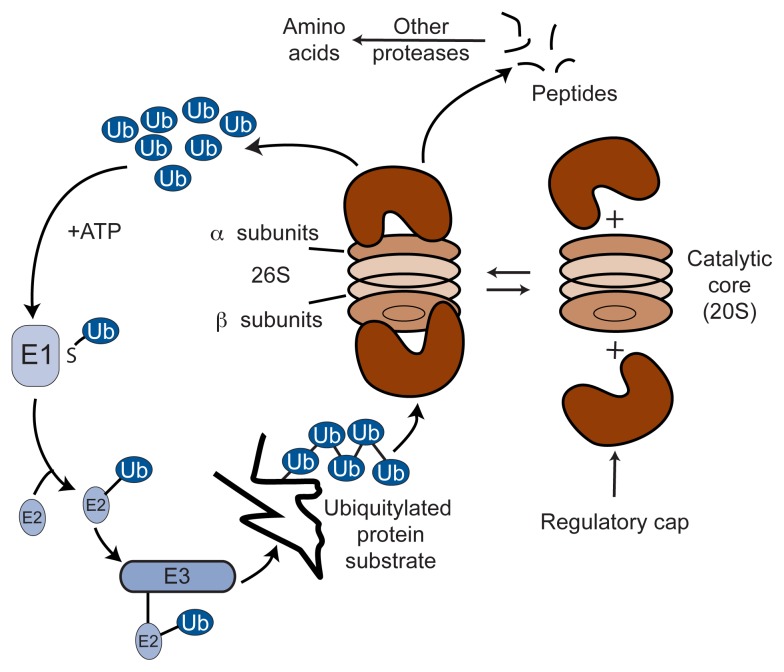
Schematic representation of the ubiquitin–proteasome system. The proteasome consists of a catalytic core and two regulatory caps. The core resembles a barrel-like structure consisting of four stacked rings that are made up of two types of subunits. One of the subunits is the site of the proteinase activities responsible for protein degradation. The two caps recognize and bind to proteins that are destined for degradation and help these proteins enter the channel in the center of the catalytic core. There, the proteins are cut into peptides that then can be degraded further into amino acids. Proteins to be degraded by the proteasome first must be marked by the addition of at least four ubiquitin molecules (Ub). Transfer of ubiquitin to the target proteins is mediated by ubiquitin-activating enzymes (E1), conjugating enzymes (E2), and ligating enzymes (E3). This process requires energy, which is provided in the form of adenosine triphosphate (ATP), the cell’s primary energy source. SOURCE: Adapted from Donohue 2002.

A functional proteasome (also called the 26S proteasome because of its relative size or weight) is composed of a smaller barrel-shaped core and two “caps” that are attached to each end of the core. The proteasome core consists of four stacked rings containing two types of subunits, all facing into a central cavity (see figure 3). These subunits together have at least five distinct proteinase activities that cleave proteins at different sites.^2^ The other major parts of the 26S proteasome are the caps at each end of the core. Each cap is a regulatory particle that also is composed of multiple subunits with numerous functions. These subunits recognize the ubiquitylated protein, cut off the ubiquitin chains from this protein, thereby “unfolding” the protein, and open the channel inside the proteasome core so that the protein can enter the channel for degradation (see figure 3).

Proteasomes are vital to all cells, and the complete absence or severe reduction of proteasome function can be lethal. For example, defective proteasome function has been found to be a key factor in the development of some human diseases, including Alzheimer’s and Parkinson’s disease, certain cancers, and alcoholic liver disease (Plemper and Hammond 2002).

#### Effects of Alcohol Consumption on the Ubiquitin–Proteasome System

The liver is the primary site of alcohol metabolism and therefore is one of the organs most likely to be damaged by heavy drinking. One effect of alcohol consumption is that it slows hepatic protein degradation by interfering with proteolytic systems, causing proteins to accumulate in liver cells. For example, as described in the previous section, alcohol impairs the synthesis and function of lysosomes. In addition, recent studies have shown that alcohol consumption also affects the ubiquitin–proteasome pathway in the liver (Donohue 2002).

**Figure 4:**
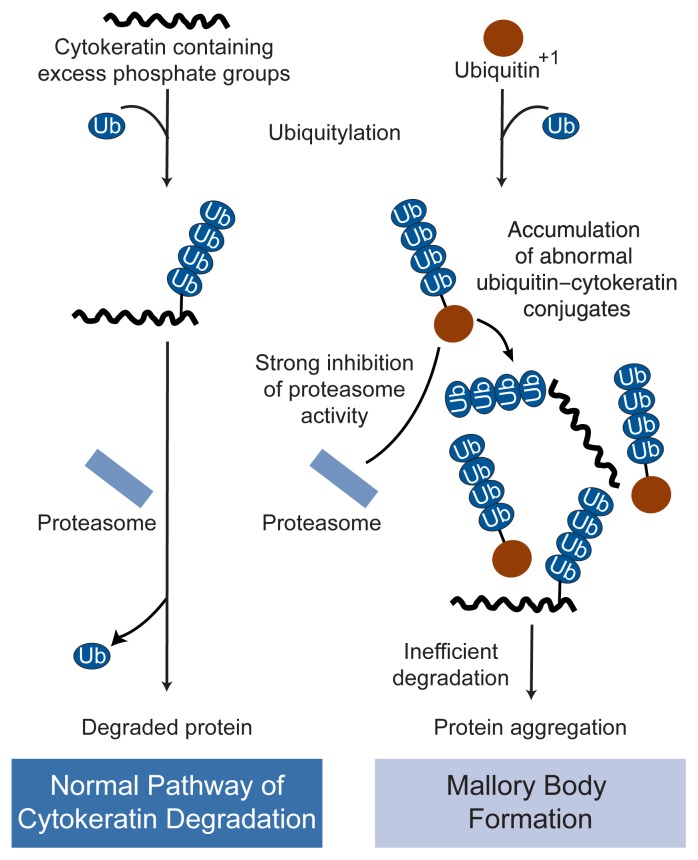
Depiction of the mechanism by which an abnormal type of ubiquitin (Ub^+1^) found in the livers of some alcoholics is thought to impair ubiquitin-mediated proteolysis of cytokeratin proteins in liver cells. The left panel shows the normal ubiquitin–proteasome pathway, in which the cytokeratin protein is modified by the addition of several ubiquitin molecules and then degraded in the proteasome. The right panel shows the modified pathway, in which Ub^+1^ forms abnormal ubiquitin–cytokeratin conjugates. Because the Ub^+1^ is difficult to remove from the cytokeratin in these conjugates, the cytokeratin cannot be degraded in the proteasome, and the Ub^+1^–cytokeratin complexes form microscopic structures called Mallory bodies. In addition, Ub^+1^ directly inhibits proteasome activity. SOURCE: Modified from McPhaul et al. 2002.

Studies have found that patients with alcohol-induced liver injury have elevated levels of ubiquitin–protein conjugates in the liquid portion of the blood (serum), with the highest levels of these conjugates found in patients with the most severe form of liver damage (i.e., alcoholic cirrhosis) (Takagi et al. 2002). Investigators have not yet identified the tissues from which these conjugates are derived; however, because the levels of the conjugates vary with the severity of liver disease, they probably have been produced in the liver. Moreover, liver cells of patients with alcoholic liver disease exhibit an accumulation of specific undegraded ubiquitin–protein conjugates called cytokeratin filaments. These proteins form microscopic structures, Mallory bodies, whose presence indicates that proteasome function is suppressed.^3^ Researchers recently demonstrated that liver cells with Mallory bodies contain an abnormal form of ubiquitin called Ub^+1^ (McPhaul et al. 2002). Ub^+1^ is formed when the gene encoding ubiquitin is “misread” by the cell’s machinery, resulting in synthesis of ubiquitin molecules that are longer than normal. When these abnormal molecules are attached to proteins, they cannot be removed as easily as normal ubiquitin, thereby slowing down the degradation of the attached protein (see figure 4). In addition, Ub^+1^ itself inhibits proteasome function and could thereby cause liver cell death. Researchers do not yet know, however, how Ub^+1^ is formed in the cells from these patients or whether alcohol plays a role in this process, because Mallory bodies also are found in patients with liver disease who do not have a history of alcohol abuse. Thus, the mechanisms underlying this novel molecular change remain to be determined.

Suppression of proteasome activity is caused not only by the presence of Ub^+1^ but also by high blood alcohol levels. For example, proteasome activity declines by up to 43 percent in certain animal models in which the animals are continuously fed alcohol, achieving blood alcohol levels that are two to three times the legal intoxicating level in humans (Donohue et al. 1998; Fataccioli et al. 1999). Although these alcohol levels exceed the alcohol concentration of 0.08 percent (i.e. 80 milligrams of alcohol per 100 milliliters of blood) that is now the legal limit of intoxication in many States, research has shown that a sizable number of apprehended drunk drivers have blood alcohol levels that are two to five times higher than the legal limit (Jones 1999). Thus, the alcohol levels used with experimental animals, while high, are in line with those recorded in humans. Similarly, researchers have found that when cultured liver cells that metabolize alcohol are exposed to comparably high alcohol concentrations, proteasome activity declines (Osna et al. 2003). Lower alcohol levels (i.e., those approximately equal to or slightly higher than 0.08 percent) do not affect proteasome activity in these cells nor do they affect the enzyme activity in animals.

The alcohol-related decrease in proteasome activity appears to be linked to the activities of two enzymes involved in alcohol metabolism—alcohol dehydrogenase and cytochrome P450 2E1 (CYP2E1). Both of these enzymes convert alcohol into acetaldehyde, a toxic and reactive substance. In addition, CYP2E1 generates highly reactive oxygen species that can inactivate proteins and contribute to liver damage (for more information on reactive oxygen species and their effects, see the article by Wu and Cederbaum in this issue). Chronic alcohol consumption can elevate the levels of (i.e., induce) CYP2E1. Under these conditions, when alcohol concentrations in the blood (and liver) reach a certain level, the alcohol can interact with CYP2E1, causing the enzyme to become resistant to degradation by the proteasome. As a result of this stabilization, CYP2E1 levels in the cell increase (Roberts et al. 1995). These elevated CYP2E1 levels can lead to excessive generation of reactive oxygen species, which in turn can inactivate the proteasome (Bardag-Gorce et al. 2000; Donohue et al. 1998; Fataccioli et al. 1999). The resulting suppression of proteasome activity can result in reduced cell viability through various mechanisms described in the following sections (also see Donohue 2002).

Excess production of reactive oxygen species and reactive molecules is one of the factors contributing to a harmful cellular state called oxidative stress. Alcohol consumption additionally contributes to oxidative stress by depleting the levels of the intracellular molecule glutathione, which acts as an antioxidant—that is, it neutralizes many of the radicals generated by CYP2E1. Therefore, alcohol contributes to oxidative stress through several mechanisms, including increased production of oxygen radicals and reduced antioxidant levels, thereby exacerbating proteasome dysfunction.

#### The Ubiquitin–Proteasome System and Alcohol-Induced Cell Death

Alcohol can cause a specific form of cell death called apoptosis. This is a form of “cell suicide” that all cells are inherently programmed to carry out in response to certain signals. The proteasome plays a critical role in regulating apoptosis and ensuring cell survival by degrading proteins that can induce apoptosis. Accordingly, suppression of proteasome function with specific inhibitors can cause cell death. In contrast, alcohol and its metabolites tend to “push” cells toward premature apoptosis through several mechanisms. For example, alcohol impairs the normal function of mitochondria—membrane-enclosed cell components in which the cell’s energy production occurs—by causing pores in the mitochondrial membrane to open, which is a signal for the cell to begin the process of apoptosis.

Alcohol-induced proteasome dysfunction also may contribute to apoptosis because proteasomes normally degrade certain proteins in the mitochondrial membrane that promote apoptosis (i.e., pro-apoptotic factors) (Naujokat and Hoffmann 2002). Therefore, one can speculate that if alcohol suppresses proteasome function, these pro-apoptotic factors could accumulate in the mitochondria and enhance liver cell apoptosis.

#### The Ubiquitin–Proteasome System and Alcohol-Induced Tissue Inflammation

Long-term alcohol consumption can cause inflammation of the liver tissue and liver cell death, leading eventually to liver injury (i.e., fibrosis and cirrhosis). The mechanisms involved in inflammation and cell death are complex, but one central cellular mechanism that underlies both these processes is the activation of a protein called nuclear factor kappa B (NF∞B). NF∞B belongs to a class of proteins called transcription factors, which bind to DNA near certain genes and enhance the “reading” (i.e., transcription) of those genes and, subsequently, the synthesis of the proteins encoded by those genes. NF∞B enhances the transcription of several genes involved in inflammation and cell death. Because it governs such potentially detrimental genes, the activity of NF∞B normally is tightly controlled. Thus, most of the time NF∞B is tethered to an inhibitor protein that prevents NF∞B from interacting with DNA. Under conditions of oxidative stress, however, a series of reactions occur that lead to the ubiquitylation of this inhibitor protein. As a result, the proteasome degrades the inhibitor protein, NF∞B becomes free to bind to DNA, and transcription of the inflammation-promoting genes can proceed. As described previously, the oxidative stress leading to this process can result from alcohol consumption. On the other hand, oxidative stress can inhibit proteasome function, as mentioned in the previous section. Researchers do not yet know exactly how these two different effects of alcohol on the proteasome system can be explained; however, it appears that after long-term (i.e., 1 to 2 months) alcohol consumption in experimental rodents (which in humans would be equivalent to about 4 years of continuous drinking), there is sufficient proteasome activity to allow NF∞B activation to proceed.

#### Summary

Excessive alcohol consumption can impair proteolysis mediated by the ubiquitin–proteasome system through several mechanisms. First, alcohol partially inactivates the proteasomes, presumably as a result of oxidative stress–related inactivation of the enzymes. Second, alcohol consumption may block ubiquitin-mediated protein degradation by promoting the generation of Ub^+1^, although the actual role of alcohol in Ub^+1^ formation is not clear. This alcohol-induced impairment of proteasome function may have profound ramifications for cell viability. For example, inhibition of proteasome activity can result in the accumulation of modified, potentially toxic proteins in cells and can cause tissue inflammation as well as premature cell death by apoptosis.

### The Calpains

The third major proteolytic system affected by alcohol consists of a family of intracellular proteinases called calpains, which require calcium for their activities. Several molecular forms of the calpains exist, but the two major ones are calpain I and calpain II. The various calpains can be distinguished by the amounts of calcium required for their activities—calpain I needs less calcium for its activity than calpain II.

The calpains are believed to be involved in several physiological processes, including the maturation and processing of certain enzymes and the breakdown of proteins associated with the cytoskeleton—a complex array of proteins that gives cells their shapes and enables them to contract and divide. Because calpains are responsible for the proteolysis of cytoskeletal proteins, investigators have suggested that calpain activity is involved in modulating cell structure in both normal and pathological states. For example, the calpains may have a major role in nerve cell development. Under pathological conditions, however, the same enzymes can become “over-activated,” resulting in extensive degradation of structural proteins and subsequent cell damage (Johnson and Guttmann 1997).

#### Effects of Alcohol Consumption on the Calpains

Studies of how alcohol consumption affects the calpains have largely been restricted to the brain. Like the liver, the brain is significantly affected by excessive alcohol consumption, which ultimately can result in alcohol-related nerve cell degeneration. Recent studies examining the effects of alcohol consumption on calpain activity in the brain demonstrated that the activity of these enzymes is elevated in the brains of alcohol-fed animals compared with untreated animals (Rajgopal and Vemuri 2002). Furthermore, the brains of alcohol-fed animals had higher levels of the degradation products of a cytoskeletal protein called spectrin, which is degraded by calpains. Researchers believe that the activation of calpain in this instance results from an alcohol-induced increase in calcium concentrations within the nerve cells (Rajgopal and Vemuri 2002). Thus, in contrast to the lysosomal and ubiquitin–proteasome systems, which are inhibited by chronic alcohol consumption, the activity of the calpain system is enhanced by chronic alcohol consumption. Excessive or untimely protein degradation, however, can be just as harmful to the organism as reduced protein degradation.

## Conclusions

This article has described the effects of alcohol consumption on the functions of three major proteolytic pathways that regulate the quantity and the types of proteins inside cells. Through a variety of mechanisms, alcohol significantly influences each of these proteolytic pathways, interfering with their normal functions. All of these changes, however, can lead to the same end results: cell death and tissue injury, which may contribute to liver disease and other physiological damage associated with chronic alcohol consumption. Although researchers have learned much about alcohol and its effects on proteolytic systems, just as many issues remain to be explored. For example, the identity of the molecules that inhibit lysosome assembly and cause proteasome inhibition has not yet been determined. The apparent paradox that NF∞B activation and the resulting inflammation occur during proteasome inhibition also requires further elaboration. Finally, researchers should investigate whether these proteolytic systems can be employed as therapeutic targets. For example, the proteasome, which is inhibited by a large number of agents, also can be activated by several small molecules, including some naturally occurring lipids (Dahlmann et al. 1993). Consequently, the administration of these compounds to alcohol-treated animals or cells may conceivably reactivate partially inactivated proteasomes and thus restore normal protein degradation after alcohol exposure.

## Footnotes

1 During endocytosis, material outside the cell is progressively enclosed by a portion of the plasma membrane, which then is taken up into the cell to form a bubble-like structure called an endosome, containing the ingested material. Endosomes then fuse with lysosomes so that the lysosomal enzymes gain access to the ingested material.

2 In immune cells, the proteasome possesses three unique subunits that replace some of the normal subunits. These three unique subunits alter the proteasome, allowing it to degrade target proteins into antigenic peptides that stimulate an immune response.

3 The process of Mallory body formation is analogous to what occurs in Alzheimer’s disease, where ubiquitylated tangles of nerve cell fibers and structures called neuronal bodies form in the brain. The formation of neuronal bodies also is related to reduced proteasome function.
